# Rice Bran Derived Bioactive Compounds Modulate Risk Factors of Cardiovascular Disease and Type 2 Diabetes Mellitus: An Updated Review

**DOI:** 10.3390/nu11112736

**Published:** 2019-11-12

**Authors:** Nancy Saji, Nidhish Francis, Lachlan J. Schwarz, Christopher L. Blanchard, Abishek B. Santhakumar

**Affiliations:** 1 Australian Research Council (ARC) Industrial Transformation Training Centre (ITTC) for Functional Grains, Graham Centre for Agricultural Innovation, Charles Sturt University, Wagga Wagga, NSW 2650, Australia; nsaji@csu.edu.au (N.S.); nfrancis@csu.edu.au (N.F.); lschwarz@csu.edu.au (L.J.S.); CBlanchard@csu.edu.au (C.L.B.); 2 School of Biomedical Sciences, Charles Sturt University, Wagga Wagga, NSW 2650, Australia; 3 School of Animal and Veterinary Sciences, Charles Sturt University, Wagga Wagga, NSW 2650, Australia; 4 School of Agricultural and Wine Sciences, Charles Sturt University, Wagga Wagga, NSW 2650, Australia

**Keywords:** bioactive compounds, cardiovascular disease, rice bran, type 2 diabetes mellitus

## Abstract

Cardiovascular disease (CVD) and type 2 diabetes mellitus (T2DM) are two chronic diseases that have claimed more lives globally than any other disease. Dietary supplementation of functional foods containing bioactive compounds is recognised to result in improvements in free-radical-mediated oxidative stress. Emerging evidence indicates that bioactive compounds derived from rice bran (RB) have therapeutic potential against cellular oxidative stress. This review aims to describe the mechanistic pathways behind CVD and T2DM development and the therapeutic potential of polyphenols derived from RB against these chronic diseases.

## 1. Introduction

The incidence of cardiovascular disease (CVD) and type 2 diabetes mellitus (T2DM) is on a steady rise and is transforming into the fastest growing chronic diseases worldwide. A compelling pathophysiological correlation exists between CVD and T2DM. Hyperglycaemia in diabetes contributes to myocardial damage, ischaemic events, and thrombosis, consequently resulting in vascular dysfunction [1,2]. Nevertheless, lifestyle-associated risk factors such as obesity, sedentary behaviour and metabolic syndrome contribute to CVD even in normoglycaemic and pre-diabetic populations. The key pathological mechanisms associated with these diseases are increased oxidative stress, inflammation, dyslipidaemia and insulin resistance [3]. Increased oxidative stress as a result of an imbalance between free radicals and natural antioxidants within the body increases the incidence of thrombogenesis, vascular inflammation and endothelial dysfunction that can, in turn, promote the progression of CVD and T2DM [4,5,6]. In this context, diet can play a pivotal role in supplying the necessary antioxidants required to combat the increasing free radical damage [7].

Rice (Oryza sativa) is a staple cereal grain consumed by approximately 3.5 billion people worldwide [8]. Rice bran (RB) is a by-product of the rice milling process; the conversion of brown rice to white rice [9]. RB is known to contain a wide range of bioactive chemicals (e.g., polyphenols), peptides (e.g., Ile-Pro) and lipids (e.g., γ-oryzanol). However, due to RB’s rich lipid content, rancidity and the associated undesirable flavours and odours develop soon after production and storage [10] and is therefore usually discarded or used as animal feed [11]. To render RB suitable for dietary consumption, stabilisation methods that can ensure the destruction of active lipases and peroxidases are often utilised [12]. Examples of such treatments include: infrared stabilisation [13], fermentation [14], enzymatic stabilisation [15], extrusion [16], microwave and conventional roasting [17]. Recent studies have demonstrated RB derived bioactive compounds display antioxidant, anti-dyslipidaemic and anti-inflammatory properties both in vitro and in vivo against CVD [18] and T2DM [19]. The primary mechanisms of action utilised by RB to elicit such disease preventive characteristics are believed to be via its antioxidant, free radical scavenging and metal chelating properties. This review aims to provide an overview of the disease preventive role of the bioactive ingredients of RB, including bioactive chemicals, proteins and fats and the mechanistic pathways by which they modulate risk factors attributed to the development of CVD and T2DM.

## 2. Rice Bran Derived Bioactive Compounds and Biomarkers of Cardiovascular Disease

The most important pathological process in cardiovascular disease (CVD) is atherosclerosis, which results in a build-up of arterial plaque that hardens over time leading to a narrowing of arteries and interference with haemostasis [4,20]. Endothelial cells that coat the inner surface of blood vessels [21] normally regulate vascular homeostasis through the release of mediators, such as nitric oxide (NO), prostacyclin (PGI2), endothelin (ET-1) and by regulating local angiotensin-II activity [22]. However, dysregulation of the homeostatic mechanisms in response to increased expression of adhesion molecules, free radicals i.e., reactive nitrogen species (RNS) and reactive oxygen species (ROS), pro-inflammatory and pro-thrombotic factors can result in endothelial dysfunction [22]. Atherosclerotic triggers can also be initiated through interaction between the endothelium, leukocytes and inflammatory mediators such as intercellular adhesion molecules (ICAM-1) and vascular adhesion molecules (VCAM-1), interleukin-1β (IL-1β), interleukin 6 (IL-6), tumour necrosis factor α (TNF-α), monocyte chemoattractant protein 1 (MCP-1), macrophage colony-stimulating factor (M-CSF), CD40 ligand (CD154), interferon γ and nuclear factor-κB (NF-κB) [23,24,25]. These signalling molecules act as secondary mediators of local vascular inflammation induced by cytokines that permit adhesion of T lymphocytes and monocytes to the arterial endothelium and their infiltration into the interior vascular surface [1]. The adhered monocytes convert to macrophages and eventually transform into lipid-rich foam cells that contribute to the formation of fatty streaks, the first morphologically distinguishable precursor of the atherosclerotic plaque [26]. As the expression of the pro-inflammatory signalling molecules continue to increase, the plaque continues to grow and ultimately destabilises and ruptures resulting in myocardial infarction or sudden death from the associated cardiac events [1,2].

### 2.1. Impact of RB Derived Bioactive Chemical Compounds

Bioactive compounds in RB are believed to target pathways involving oxidative stress, thrombogenesis, and vascular inflammation via their antioxidant activity. The presence of acylated steryl glucosides [27], flavonoids [28], ferulic acid [29], policosanol [30], resveratrol [31] and plasma metabolites [32] from RB have been demonstrated to exhibit antioxidant, anti-inflammatory and free radical scavenging properties consequently instrumental in preventing or delaying the occurrence of CVD.

Metabolomic analysis on rice demonstrated the presence of bioactive flavonoids, such as tricin and its isomers, tricin 7-O-rutinoside and tricin 7-O-β-d-glucopyranoside, to reside mainly in the leaf and RB [28]. It was also observed that tricin 4′-O-(erythro-β-guaiacylglyceryl) ether and isoscoparin 2″-O-(6‴-(E)-feruloyl)-glucopyranoside found in RB displayed the most potent activity for inhibiting NO production and 1,1-diphenyl-2-picrylhydrazyl (DPPH) radical-scavenging activity, respectively. Feruloylated oligosaccharides (bound form of ferulic acid) extracted from RB have also been recognised to suppress atherosclerotic triggers, such as TNF-α, IL-1β, IL-6 and NO and activation of IL-10 production in lipopolysaccharide (LPS) stimulated macrophage (RAW264.7) cells in a dose-dependent manner (0.1–100 μg/mL) [29]. Additionally, prostaglandin E2 (PGE 2) production, typically observed in vascular inflammatory responses, was shown to be suppressed by feruloylated oligosaccharides (100 μg/mL) [29]. Policosanol extracted from RB wax was demonstrated to exhibit distinct anti-thrombotic activity [30]. Rat platelets pre-treated with various concentrations (125–1000 μg/mL) of policosanol extract resulted in reduced platelet adhesion to collagen and cellular protein secretion in a dose-dependent manner [30].

RB supplementation has been demonstrated to have considerable cardioprotective effects in vivo animal models (Table 1). Apolipoprotein E (ApoE^−/−^) knockout mice (ApoE protein-deficient mice) serves as a suitable model to study CVD because when the normal function of this protein is disrupted, it results in an accumulation of cholesterol ester-enriched particles in the blood, consequently leading to the development of atherosclerotic plaques [33]. A study conducted by Perez-Ternero, Claro [34] has reported that supplementation of 1% or 5% RB enzymatic extract to ApoE^−/−^ knockout mice can reduce plaque development and liver steatosis via attenuation of the 3-hydroxy-3-methylglutaryl-coenzyme A (HMG-CoA) reductase pathway. Moreover, Perez-Ternero, Claro [34] also demonstrated the reduction of lipid deposition, macrophage infiltration in the aortic sinus and down-regulation of ICAM-1 and VCAM-1 expression in ApoE^−/−^ knockout mice. Similarly, another study conducted by Perez-Ternero, Herrera [18] reported that RB enzymatic extract lowered cholesterol and prevented atherosclerotic plaque development in ApoE^−/−^ knockout mice. Since ApoE protein deficiency results in an inability to transport lipids such as fats and cholesterol in the blood, an increase in the risk of plaque formation leading to atherosclerosis occurs as a result. However, these studies were able to show that supplementation with RB enzymatic extract can potentially help reduce plaque development and ameliorate CVD development.

Furthermore, beneficial actions of RB enzymatic extract on dyslipidemia, hyperinsulinemia and hypertension in obese rats supplemented with either 1% or 5% RB enzymatic extract for over 20 weeks, demonstrated a restoration of microvascular function through a noticeable increase in NO, endothelial nitric oxide synthase (eNOS) and expression of calcium-activated potassium channels [35]. Substantial alleviation of O_2_^−^ production and microvascular inflammation [35], inducible nitric oxide synthase (iNOS) expression, TNF-α expression and nicotinamide adenine dinucleotide phosphate (NADPH) oxidase regulation was observed after obese Zucker rats were fed a 1% or 5% RB enzymatic extract supplemented diet [36]. The RB enzymatic extract diet also ameliorated endothelial dysfunction and vascular hyperreactivity to phenylephrine by the up-regulation of eNOS protein expression [36]. An in vivo study by Mukherjee, Ray [31] investigated the effect of RB derived bioactive compounds when combined with other bioactive compounds in improving cardiac function. Male Sprague-Dawley rats supplemented with 10 mg/kg/day of Longevinex (resveratrol supplement containing 5% quercetin and 5% RB derived phytate) for one and three months exhibited superior cardiac performance (improved aortic flow and left ventricular function), reduced myocardial infarct size and demonstrated an induction of survival signals [31]. This was evidenced by the increased expression of anti-apoptotic protein Bcl-2 and reduced expression of pro-apoptotic protein Bax, enhanced Akt phosphorylation by signalling the PI-3-kinase–Akt pathway and increased the expression of sirtuin proteins, SIRT1 (nuclear) and SIRT3 (mitochondria), that have been linked with longevity [31]. The mechanisms described in this paper were directly correlated with improved cardiac performance. Hence, RB derived bioactive compounds when combined with other bioactive compounds may be a useful strategy that could be used to improve overall cardiac function.

Although RB is an excellent source of several antioxidant compounds including γ-oryzanol and ferulic acid as described by previous studies, their bioavailability and metabolism within the body are uncertain and require further investigation. Perez-Ternero, Macià [37] conducted a study to examine the oral bioavailability and metabolic pathways in a Wistar rat model where the rats were administered with RB enzymatic extract and sacrificed over a period of 24–48 h to obtain plasma, urine and faecal samples. After RB enzymatic extract consumption, total recovery of ferulic acid accounted for 18.8% of the dose administered, possibly owing to hydrophobic interactions between proteins and γ-oryzanol resulting in improved solubility. Upon examination of the plasma and urine samples, 25 ferulic acid metabolites were observed to have displayed a biphasic response (absorption and metabolisation). In plasma, the presence of ferulic acid in its sulphate and glucuronide forms was reported while dihydroferulic acid generation from ferulic acid was reported in the colon owing to interactions with the large intestine microflora. Most of the metabolites found in plasma were also identified in urine, indicating a fast metabolisation rate. After analysis of the faecal matter, colonic metabolism was found to have led to the production of simpler phenolic compounds. Moreover, O_2_^−^ production was observed to have been annulled by phenolic compound enriched plasma, thus showing the biological potential of RB enzymatic extract as a nutraceutical ingredient [37]. The results obtained in this study appear to suggest that ferulic acid, in the form of ferulic acid-derived metabolites, is the main antioxidant molecule in RB enzymatic extract. However, ferulic acid is rapidly metabolised, giving rise to multiple metabolites that maintained the inhibitory activity upon NADPH oxidase complex. One of the limitations associated with this study was that since most of the ferulic acid metabolites identified in plasma are not commercially available, the individual effects of the metabolites generated were not able to be examined.

Human clinical studies have reciprocated similar findings as previously highlighted with in vitro and in vivo animal studies (Table 1). A prospective cohort study conducted on whole-grain consumption and its relation to ischemic stroke by Juan, Liu [38], in which 71,750 women and 42,823 men participated, established that a higher intake of whole grains and bran can potentially lower the risk of CVD related diseases. In a dietary intervention study conducted on 38 children (10 ± 0.8 years) with abnormal cholesterol levels, significant differences in plasma lipid metabolites after consumption of RB [32] were observed. Consumption of RB alone significantly increased metabolites, such as sphingolipid, ceramide, the secondary bile acid glychodeoxycholate sulfate, as well as several phospholipids and monohydroxy fatty acids. After consumption of RB combined with navy bean, a significant increase in lipid metabolites including lysolipid, phospholipids and endocannabinoid N-oleoyltaurine, and a decrease in carnitine was observed [32]. Moreover, levels of diet-derived amino acids, bioactive chemicals (particularly salicylate) and cofactors/vitamins (e.g., pyridoxal, an active form of vitamin B6) increased compared to the control after RB consumption [32]. In another study, pre-germinated RB extract containing acylated steryl glucosides (PSG) (6 capsules/day containing 50 mg of PSG) was found to significantly reduce serum low-density lipoprotein (LDL) and TNF-α levels [27]. These results demonstrate that consumption of RB and navy bean can impact blood lipid metabolism with implications for reducing CVD risk in children.

In summary, metabolome analysis of RB has revealed the presence of bioactive flavonoids such as tricin, its isomers and feruloylated oligosaccharides to suppress several atherosclerotic triggers. In several animal studies, supplementation of RB enzymatic extract was observed to reduce plaque development, provide improvements in dyslipidemia, hyperinsulinemia and hypertension. Consumption of RB derived compounds in combination with other bioactive substances such as longevinex exhibited superior cardiac performance reduced myocardial infarct size and demonstrated induction of survival signals. Notably, there were a few studies involving humans. A prospective cohort study on whole-grain consumption and incidence of ischemic stroke established that higher intake of whole grains and bran can potentially lower the risk of CVD related diseases. In children with abnormal cholesterol levels, significant differences in plasma lipid metabolites after consumption of RB was observed. Although RB is an excellent source of several bioactive compounds, their bioavailability and metabolism in vivo require further investigation.

### 2.2. Impact of RB Derived Bioactive Peptides

RB is also known to contain physiologically active peptides (Table 1) that exert various biological properties including antioxidant, angiotensin-1-converting enzyme (ACE) inhibitory activity and restoration of vascular inflammation and endothelial function (Boonla et al., 2015; Senaphan et al., 2018). RB derived bioactive peptides have been shown to alleviate oxidative injury to human umbilical vein endothelial cells (HUVEC) under H_2_O_2_ induced oxidative stress conditions [39]. Cell morphology changes as a result of H_2_O_2_ damage being ameliorated and cell apoptosis was significantly lowered through the attenuation of cleaved caspase-3 levels as observed post RB peptide treatment [39]. A similar effect was demonstrated in vivo after RB protein hydrolysate supplementation in atherosclerosis-prone rats fed with a high-carbohydrate and high-fat diet [40]. Significant improvements to levels of hyperglycemia, insulin resistance, dyslipidemia, hypertension, aortic pulse wave velocity, aortic wall hypertrophy and vascular remodelling was observed [40]. Another investigation using hypertensive rats supplemented with RB protein hydrolysates demonstrated increased endothelium-dependent vasorelaxation, eNOS protein, and plasma nitrate/nitrite levels and a decrease in blood pressure, peripheral vascular resistance, plasma ACE, O_2_^−^ formation, plasma malondialdehyde and p47phox protein [41]. The results from these studies suggested that RB derived bioactive peptides possess anti-hypertensive properties that can help restore endothelial function and hemodynamic status as a result of a reduction in oxidative stress.

### 2.3. Impact of Rice Bran Oil

The use of RB oil as a specialty cooking oil is becoming increasingly popular among consumers due to its purported free radical scavenging, anti-inflammatory, antidyslipidaemic, and anti-thrombotic properties (Table 1). Such effects have been observed in esterified RB oil wherein a significant increase in DPPH radical scavenging activity and anti-inflammatory potential was observed via suppression of iNOS and cyclooxygenase (COX)-2 mRNA expression in a dose-dependent manner in LPS stimulated RAW264.7 macrophage cell line [42]. It has been suggested that the observed effect could be attributed to an increase in the ferulic acid and γ-oryzanol content during the esterification reaction. Similarly, in another in vitro study involving LPS induced ROS production in RAW264.7 cells, γ-oryzanol treatment was noted to COX-2 expression by suppressing Erk1/2-mediated Egr-1 expression [43], providing further evidence to the beneficial properties of γ-oryzanol present in the RB.

Rao, Sugasini [44] demonstrated that the unsaponifiable fraction of RB oil significantly contributes to its anti-inflammatory activity by targeting the NF-κB pro-inflammatory signalling pathway. Pro-inflammatory mediators like ROS (O_2_^−^ and NO), eicosanoids (PGE2, TXB2, LTB4 and LTC4), cytokines (TNF-α and IL-6) and hydrolytic enzymes (collagenase, elastase and hyaluronidase) were notably reduced in peritoneal macrophages isolated from male Wistar rats fed a crude RB oil diet containing unsaponifiable fraction [44]. To produce high-quality edible oil, a physical refining process is conducted to remove by-products, such as gums, waxes and free fatty acids via distillation that is otherwise generally found in crude RB oil. Male Wistar rats fed a physically refined RB oil diet resulted in increased secretion of anti-inflammatory mediators (IL-4 and IL-10) [44]. Therefore, to promote cardiovascular health and to curtail pro-inflammatory mediators, consumption of physically refined RB oil with added γ-oryzanol content is more beneficial than mere consumption of crude or refined RB oil [44]. Ausman, Rong [45] also demonstrated several beneficial properties of physically refined RB oil diet in hamsters that included a significant decrease in hepatic HMG-CoA reductase activity (an enzyme of the mevalonate pathway that produces cholesterol and other isoprenoids), aortic fatty streak, total plasma cholesterol, cholesterol absorption and LDL. In addition, an increase in intestinal HMG-CoA reductase and neutral sterol excretion with no effect on bile acid excretion was also observed [45]. Francisqueti, Minatel [46] investigated the therapeutic effect of γ-oryzanol derived from RB oil in the prevention of cardiorenal metabolic syndrome wherein Wistar rats were subjected to a high sugar-fat diet for 20 weeks supplemented with or without γ-oryzanol. The diet consisting of γ-oryzanol supplementation also prevented weight gain, hypertriglyceridemia, systolic, and diastolic dysfunction [46].

Furthermore, the anti-thrombotic potential of RB oil has also been investigated in an in vivo study using a rodent model. Supplementation of interesterified and blended oil of coconut/RB or coconut/sesame oil in male Wistar rats showed a significant decrease in the rate of adenosine diphosphate (ADP) and collagen-induced aggregation of platelets and an increase in the prostacyclin/thromboxane ratio in rat serum [47]. Since platelet aggregation is a critical incident in thrombus formation, the anti-thrombotic effect of RB oil supplementation can have extensive involvement in amelioration of CVD pathological processes.

In humans, when the effect of dietary supplementation of palm oil, RB oil and coconut oil was examined in normolipidaemic subjects, increased susceptibility to develop exaggerated chylomicron (small fat globules that transport lipids to tissues) remnantaemia were reported. This was in response to meals enriched in saturated fatty acids derived from a palm oil diet, thus contributing to increased atherogenic risk [48]. However, individuals consuming an RB or coconut oil diet did not develop atherogenic risk factors [48]. In hyperlipidemic patients, an RB oil supplemented diet reduced weight, body mass index, waist and hip circumferences [49], total cholesterol, LDL and the atherogenic ratio of total cholesterol/HDL [49]. Similarly, Kuriyan, Gopinath [50] also confirmed that RB oil when compared to other conventional cooking oils such as sunflower oil, significantly reduced plasma total cholesterol and triglyceride levels in hyperglycemic patients. Furthermore, blending RB oil with other domestic cooking oils such as safflower oil [51,52] or sesame oil [53] has also displayed several cardioprotective properties. A reduction in oxidized LDL, total cholesterol and high sensitivity C-reactive proteins was observed in hyperlipidemic patients after consuming safflower and RB blended oil for three months [51]. Similarly, a study by Malve, Kerkar [52] explored the effects of substitution of domestic cooking oils with blended RB and safflower oil (80:20) for three months and identified that 82% of patients from this group had LDL levels less than 150 mg/dL. Similarly, consumption of sesame and RB blended oil for 60 days resulted in a reduction in blood pressure, hyperglycemia and improvements in the lipid profile in hypertensive subjects compared to normotensive subjects [53]. Therefore, results from all these human dietary intervention studies suggest that the use of blended oils at different proportions as a substitute to a single conventional cooking oil is predicted to be another promising strategy that may help modulate hypertension linked CVD.

In addition to the demonstrated favourable bioactive actions associated with RB, there are practical implications associated with polyphenol interactions in biological systems that need to be considered. Due to the absence of free radicals such as DPPH in the human body, and the complexity associated with the mechanism of action, there are several criticisms related to studies involving in vitro assays. Although a number of in vitro studies have demonstrated significant favourable biological responses in vitro via its potential antioxidant activity, this cannot necessarily translate in vivo. For example, in one of a widely used antioxidant assay (ferric reducing antioxidant power), the reaction is performed at acidic pH 3.6, suggesting that the data obtained cannot be used as anything more than a simple screening method to determine potential antioxidant capacity [54]. Furthermore, although the in vitro studies demonstrate the capability of RB to provide cardioprotective effects, the identity of the specific compounds within RB that contribute to the observed impacts remains unknown. Moreover, it is also to be noted that minimal studies have been conducted that have investigated the bioavailability of bioactive compounds in RB in vitro or in vivo models. 

## 3. Rice Bran Derived Bioactive Compounds and Biomarkers of Type 2 Diabetes Mellitus

Oxidative stress and inflammation play essential roles in the pathogenesis of T2DM through the development of β cell dysfunction and insulin resistance, subsequently resulting in hyperglycaemia [3]. Excess glucose and fatty acids build-up within muscles, adipose tissue and pancreatic cells combined with a sedentary lifestyle normally lead to the excess production of ROS and RNS [55]. A balance between free radical generation and scavenging is crucial at a cellular level, and any imbalances may result in an oxidative stress environment that alters insulin sensitivity by either increasing insulin resistance or impairing glucose tolerance via several cell signalling pathways [56]. Risk factors associated with T2DM include obesity, high blood pressure (hypertension) and dyslipidaemia, which is caused as a result of elevated plasma cholesterol, triglycerides or LDL levels.

The underlying pathophysiological mechanisms of T2DM and related metabolic disorders are attributed to the generation of ROS/RNS via activation of advanced glycation end-products (AGE), polyol flux, hexamine and protein kinase C (PKC) pathways [57,58]. AGEs are formed as a result of glycol-oxidation results in activation of surface receptors that contribute to vascular injury and endoneurial hypoxia [59]. An increase in cellular glucose results in the activation of the polyol pathway, which results in reduced NADPH dependent antioxidants like glutathione (GSH) that further intensifies cellular stress [60]. Cellular stress leads to the development of insulin resistance, β-cell dysfunction, impaired glucose tolerance and eventually T2DM. Insulin resistance may also be induced by many cell signalling proteins involved in systemic inflammation, such as TNF-α, IL-1β and interferon-γ resulting in hyperglycaemia [60]. A cellular upsurge of glucose or free fatty acids (FFA) results in the activation of the hexosamine pathway which leads to overexpression of transforming growth factors such as TGF-α and TGF-β and plasminogen activator inhibitor-1 (PAI-1) that further contributes to diabetes-related disorders [61,62]. Furthermore, under the adverse effects of chronic hyperglycemia, the hexosamine pathway plays a key role in insulin resistance and also leads to β-cell dysfunction under oxidative stress conditions [63]. Hyperglycemia in cells stimulates the production of di-acyl glycerol (DAG) that activates multiple PKC isoforms and further stimulates the expression of other signalling pathways such as PAI-1, NF-κB and TGF-β known to be involved in diabetic caused metabolic disorders [64,65].

### 3.1. Impact of RB Phenolics and Other Chemical Compounds

Bioactive compounds extracted from RB have been demonstrated to favourably modulate pathways associated with hypertension, hyperlipidaemia and hyperglycemia, the major precursors to the pathophysiology of T2DM (Table 2). A review of the literature reported two relevant in vitro studies that demonstrate the anti-diabetic potential of RB phenolics. RB extract displayed a dose-dependent (0.032, 0.1, 0.32 and 1.0 mg/mL) effect on insulin release in INS-1 cells [66]. The responses observed may be owing to a synergistic effect of several compounds present in RB extract including policosanol and γ-oryzanol, known for its ability to lower cholesterol levels via modulating HMG-CoA reductase activity [66]. RB derived phenolic compounds (41.65 mg of catechin equivalents per 100 g of defatted RB) extracted using ethyl acetate was found to be a robust α-glucosidase inhibitor compared to purified ferulic acid in vitro [67]. Since α-glucosidase is the major enzyme responsible for breaking down starch and disaccharides to glucose, the digestibility of starch may be lowered using RB extract, possibly due to a synergistic effect of various phenolic compounds present in the RB ethyl acetate fraction.

Further examinations in vivo involving consumption of RB derived phenolic acid fraction (0.2 g/kg) resulted in a considerable reduction in blood glucose levels, total cholesterol and LDL and increased plasma insulin levels, hepatic glycogen synthesis and glucokinase activity in diabetic C57BL/KsJ-db/db mice [67]. A similar study compared the effect of RB extract (10 mg/kg) and γ-tocotrienol (10 µg/kg) in rats [66]. Results from this study demonstrated that RB extract increased plasma insulin levels but did not affect blood glucose levels and purified γ-tocotrienol reduced blood glucose levels without affecting insulin release. Although γ-tocotrienol is known to activate genes involved in lipid metabolism (i.e., PPAR-γ), the lack of an acute anti-diabetic effect observed in this study suggests that γ-tocotrienols may have a long-term impact rather than an immediate response. A study by Ardiansyah, Shirakawa [68] compared how two different RB extraction procedures may affect the outcome in hypertensive rats. Upon the characterisation of these RB fractions, the phenolic contents of driselase (a cell wall degrading enzyme) and ethanol fractions were found to be 64.74 and 139.60 mg/g gallic acid equivalent, respectively [68]. In hypertensive rats, after supplementation with 60 g/kg of driselase and ethanol fractions of RB, a significant decline in blood pressure, blood urea nitrogen/creatinine ratio, albumin, total cholesterol, triglyceride, glucose levels, HDL/LDL ratio, ACE inhibitory activity, NO and urinary 8-hydroxy-2′-deoxyguanosine was observed [68]. A similar in vivo study comparing driselase-treated fraction of RB to purified ferulic acid also reported comparable outcomes [69]. A driselase RB diet supplemented at 60 g/kg significantly reduced blood pressure, albumin, glucose, insulin, incremental area under the curve glucose, blood urea nitrogen/creatinine ratio, mRNA expressions of several metabolic parameters involved in glucose and lipid metabolisms, plasma total cholesterol, triacylglycerol, ACE inhibitory activity and NOx levels [69]. Further examinations of the driselase fraction of RB demonstrated the active component responsible for lowered blood pressure and blood glucose levels to be adenosine [70]. After chronic consumption of purified adenosine solution (10 mg/L), stroke-prone spontaneously hypertensive rats showed a significant decrease in plasma LDL, blood pressure, total cholesterol, triglyceride, FFA, glucose, blood urea nitrogen/creatinine ratio, albumin, leptin, hepatic total lipid, total cholesterol and triglyceride compared to the control group [70].

Research has also investigated the use of fermented RB as an anti-hypertensive and anti-diabetic supplement. Alauddin, Shirakawa [71] investigated the effectiveness of a dual fermented RB prepared using fungi and lactic acid bacteria in stroke-prone spontaneously hypertensive rats. After an acute (single oral dose) administration of fermented RB (2 g/kg body weight), a significant decrease in systolic blood pressure, plasma glucose and insulin levels were observed [71]. This study further investigated the effects of chronic supplementation (fouor weeks) of fermented RB on these hypertensive rats which resulted in a decrease in blood pressure, weight gain, epididymal fat mass, glucose, insulin, total cholesterol, triglyceride, LDL, leptin/adiponectin ratio and hepatic parameters (total lipid, total cholesterol and total triglyceride levels). In addition, chronic supplementation significantly lowered the expression of genes regulating gluconeogenesis and lipogenesis in the liver, such as glucose-6-phosphatase catalytic subunit, phosphoenolpyruvate carboxykinase and fatty acid synthase [71]. The ability of fermented RB to reduce transcription factors involved in hepatic glucose and fat metabolism may be a strategic approach to preventing diseases involving insulin resistance and hyperlipidemia. A human clinical trial performed on T2DM patients demonstrated that the consumption of stabilised RB reduced postprandial glucose, HbA1c, total cholesterol, LDL, plasma FFA levels and increased adiponectin levels compared to control patients who consumed milled rice flour sprinkled on their food or drink [72]. The significant effects observed in this study is possibly due to higher levels of dietary fibre, oil, γ-tocotrienol, α- tocotrienol and γ-oryzanol found in RB that act synergistically for the desired outcome.

In summary, RB derived phenolic extract has been shown to significantly reduce blood glucose levels, total cholesterol and LDL, and increase plasma insulin levels, hepatic glycogen synthesis and glucokinase activity through in vitro and in vivo studies. Studies have also demonstrated RB to be a potent α-glucosidase inhibitor, suggesting the potential use of RB phenolic extracts as a dietary supplement in T2DM patients. Investigations on fermented RB supplementation in hypertensive rats provided further evidence on the anti-diabetic potential of RB that could be augmented through the process of fermentation. Based on the in vitro and in vivo investigations, it is very likely that the anti-diabetic effects after RB supplementation are a synergistic effect of various compounds present in RB including polyphenols, tocotrienol, oryzanol and unidentified compounds.

### 3.2. Impact of RB Bioactive Peptides

Rice bran contains several bioactive peptides with protease inhibitory property and can regulate the activity of circulating peptide hormones such as glucagon-like peptide-1 (GLP-1), an insulinotropic gut hormone released in response to nutrient ingestion [73,74]. Human dipeptidyl peptidase IV (DPP-IV) is a serine protease that modulates the biological activity of GLP-1. DPP-IV inhibition can, therefore, prevent cleavage of active GLP-1, thereby allowing active GLP-1 to exert its insulinotropic action. Thus, inhibitors of DPP-IV have the potential to be used as therapeutic drugs to control postprandial glycaemia in T2DM [73]. In the study conducted by Hatanaka, Inoue [73], RB bioactive peptides were generated using two commercially available proteases, namely Umamizyme G and RBioprase SP. Umamizyme G protease resulted in a higher RB peptide yield and was 11 times more effective in the inhibition of DPP-IV when compared to Bioprase SP in vitro [73]. Using HPLC and LC-MS analysis, the specific amino acids responsible for DPP-IV inhibitory activity was found to be Leu-Pro and Ile-Pro at a quantity of 1.69 and 1.22 μg/mg, respectively [73]. This study shows that proteases found in RB extract could lead to DPP-IV inhibition which can prevent GLP-1 cleavage, sanctioning GLP-1 to employ its insulinotropic activity, thus demonstrating the anti-diabetic potential for RB.

To examine the effect of RB protein hydrolysates on insulin resistance, Boonloh, Kukongviriyapan [19] utilised HepG2 cells in which insulin resistance was induced with either IL-6 or high glucose, and the regulation of insulin signalling was examined after treatment with different concentrations of RB protein hydrolysates (400–1600 μg mL^−1^). From the data obtained, an improved glucose utilization and AMPK phosphorylation and decreased Akt phosphorylation, suppressor of cytokine signaling 3 (SOCS-3) expression and janus kinase (JAK) expression ultimately resulting in the suppression of IL-6-induced insulin resistance was observed. Following on, this study also undertook an in vivo study where rats fed with a high carbohydrate-high fat diet were gavaged with 100 or 500 mg/kg of RB protein hydrolysates. The body weight and retroperitoneal fat were found to be reduced in the RB protein hydrolysate supplemented group when compared to the control. In addition, blood glucose and lipids (total cholesterol, LDL and triglycerides) levels were also found to be significantly reduced upon RB protein hydrolysate supplementation. Furthermore, RB protein hydrolysate treatment increased IL-10 expression, adiponectin levels, expression of peroxisome proliferator-activated gamma (PPAR-γ) in adipose tissues and suppressed the levels of leptin, expression of lipogenic genes (SREBF1 and FASN) and mRNA levels of proinflammatory cytokines such as Il-6, TNF-α, NOS-2 and MCP-1 [75]. Thus, the overall results from this study highlight the multiple potential benefits (anti-diabetic, anti-obesity and anti-inflammatory) of RB peptide hydrolysates.

Administration of enzymatically treated RB extract could be another suitable treatment for alleviating obesity-derived metabolic disorders, including hyperlipidemia, hypertension and hyperinsulinemia. Supplementation of RB enzymatic extract demonstrated a significant reduction in HOMA-IR index, pro-inflammatory values of NO, circulating levels of triglycerides, total cholesterol and increased HDL and adiponectin levels without altering non-esterified fatty acids in obese Zucker rats [76]. Justo, Rodriguez–Rodriguez [76] suggested that these effects could be owing to the synergistic effect of nutraceutical compounds present in RB, including peptides, free amino acids, γ-oryzanol, ferulic acids, phytosterols, and tocotrienols. A similar in vivo study was conducted by Candiracci, Justo [77] where they reported that consumption of 1% or 5% RB enzymatic extract in obese Zucker rats substantially reduced body weight and the production of oxidative stimulants such as TNF-α, IL-6, IL-1β, iNOS, and NO. These findings evidence the nutraceutical properties of enzymatic RB extract against the pathogenesis of metabolic syndrome by attenuating dyslipidemia, hypertension and insulin resistance in addition to restoring hypoadiponectinemia associated with obesity.

A review of the literature has reported a limited number of human clinical trials that demonstrate RB anti-diabetic potential. One such trial involved an experiment on healthy, Chinese males where the effect on the glycaemic index (GI) was examined [78]. They found that the co-ingestion of carbohydrates (in the form of white bread) with RB-laced soymilk and sugar-free soymilk can lower the GI by stimulating early-phase insulin secretion and thereby increasing the efficiency of blood glucose clearance due to an increased dose of protein [78]. A possible mechanism by which GI was lowered may include delayed gastric emptying as it slows down glucose release into the intestine, resulting in reduced glucose absorption into the systemic circulation, leading to a reduced glycemic response [78]. This implies that the consumption of RB and its components may offer therapeutic potential for glycemic and insulinemic control.

Taken together, several studies both in vitro and in vivo have identified RB bioactive peptides to provide antioxidant, anti-obesity and anti-hypertensive benefits [66,67,68,70,71,73,74]. It is worth mentioning that most of the studies that characterised RB protein hydrolysate extracts revealed that hydrolysates, although composed primarily of protein, also contained other components such as fibre, fat and flavonoids. Therefore, these observed effects are possibly due to a synergistic effect of various bioactive components, including peptides, free amino acids, γ-oryzanol, ferulic acids, phytosterols and tocotrienols present in RB. Although RB serves as a great source of several bioactive peptides, their bioavailability and metabolism within the body require further investigation.

### 3.3. Impact of Rice Bran Oil

Consumption of RB oil is identified to exhibit protection against hyperlipidaemia and hyperinsulinemic responses due to the presence of bioactive compounds such as γ-oryzanol and tocotrienol in RB oil [79]. Therefore, improvements in the lipid profile through RB oil dietary supplementation may be a strategic approach to indirectly improve the prognosis for T2DM. Several in vivo studies provide evidence for the potential use of RB oil in T2DM [79,80,81]. When diabetic rats were supplemented with a RB oil diet (10 or 15 g), a marked reduction in insulin/glucose ratio, LDL, non-esterified fatty acid, triglycerides, total cholesterol and fatty acids (stearic acid, arachidic acid, linoleic acid, linolenic acid and eicosapentaenoic acid) were observed by Chen and Cheng [80]. In addition, RB oil diet supplementation also resulted in improvements in faecal neutral sterol, bile acid excretion and mRNA expression of enzymes associated with cholesterol metabolism, namely hepatic cholesterol 7α-hydroxylase, LDL-receptor and HMG-CoA reductase [80]. These observed effects on improvements in lipid profile and insulin response may largely be due to the presence of γ-oryzanol and γ-tocotrienol in the RB oil diet as used in the current study. A similar study by Chou, Ma [79] provided further evidence for the hypocholesterolemic effect in T2DM rats upon supplementation with 15% RB oil. The RB oil used in this study was characterised to contain 5.25 g γ-oryzanol and 0.9 g γ-tocotrienol/150 g of RB oil. RB oil supplementation reduced total cholesterol/HDL ratio, non-esterified fatty acids, area under the curve for insulin and hepatic cholesterol. Moreover, the RB oil supplemented group had a higher HDL concentration, greater excretion of faecal neutral sterols/bile acid, total SFA and MUFA in plasma and liver signifying that RB oil may improve lipid abnormalities, diminish atherogenic index and subdue the hyperinsulinemic response in T2DM rats [79]. Therefore, improvements in cholesterol using RB oil may be a major health strategy that can be utilised to help ameliorate T2DM development.

To examine the effect of native and minor constituent-removed oils, Wistar rats were placed under an AIN-93 diet supplemented with 10 wt% of groundnut, RB and sesame oil. After RB and sesame oil consumption, a decline in lipids, 8-hydroxy-2-deoxyguanosine, hepatic cytokines and eicosanoids in leukocytes was observed [81]. While consumption of native RB and sesame oil resulted in an up-regulation of SREBP-2, PPAR-γ and down-regulation of NF-κB p65, consumption of minor constituents of RB and sesame oil resulted in a modulation of lipid homeostasis and inflammatory markers, compromising the effect of native oils [81]. This study shows a clear correlation between minor constituents of RB and sesame oil and hypolipidemic and anti-inflammatory properties, which may help reduce the likelihood of acquiring T2DM.

Human clinical trials provide further evidence on the positive impact of RB oil in maintaining lipid homeostasis and normal blood glucose levels. An investigation on T2DM patients subjected to RB dietary intervention (250 mL of modified milk daily containing 18 g of RB oil) significantly increased glucose parameters (fasting, 2-h postprandial blood glucose concentrations and the area under the curve for postprandial plasma glucose), however, total serum cholesterol and LDL concentrations decreased significantly [82]. Lai, Chen [82] have stated that this may be due to the presence of a higher MUFA or palmitic acid quantity in RB oil group compared to soybean oil (placebo group) which is known to affect plasma glucose and insulin sensitivity via regulation of TNFα and resistin gene. In another human clinical study where postmenopausal women with T2DM (*n* = 72) consumed 30 g of domestic cooking oils (canola, RB oil or sunflower oil) daily as part of a balanced diet for a period of eight weeks, a reduction in serum total cholesterol, triglyceride and LDL in both canola and RB oil treatment group compared to the sunflower control group was observed [83]. One of the underlying mechanisms that account for the cholesterol-lowering ability of RB oil may be due to its γ-oryzanol content that increases the excretion of bile acids. An increased concentration of bile acids decreases the intestinal absorption of cholesterol and increases its excretion in the faeces [83]. The overall results from this study suggest that RB oil improves lipid profile very efficiently and attenuates lipid disorders in T2DM women.

In summary, RB derived bioactive proteins and peptides are recognised to target genes involved in lipid and glucose metabolism resulting in T2DM amelioration via improvements in hypertension, hyperlipidemia and hyperglycemia (Table 2). The effects observed may be owing to a synergistic effect of unsaponifiable components, including γ-oryzanol and γ-tocotrienol found in RB oil. Human clinical trials examining the effects of RB oil on blood lipid profiles and insulin resistance in T2DM participants showed a significant decrease in several parameters, including total serum cholesterol after consuming RB oil providing further evidence to the efficacy of RB oil in ameliorating T2DM. However, since there is minimal human in vivo studies, information regarding bioavailability is limited, and this appears to be the gap in the literature concerning the anti-diabetic effect of RB derivatives.

## 4. Bioactive Compounds: Hormesis and Bioavailability

Although RB derived bioactive compounds have been demonstrated by several in vitro, ex vivo and in vivo studies to exhibit disease preventive properties, there are a number of physiological factors that need to be considered. One of the primary concerns associated with bioactive compounds in eliciting a biological response in vivo is its bioavailability. The chemical structure of polyphenols plays an important role in determining the extent of their absorption in the intestinal epithelia and the nature of its plasma metabolites. A shortened half-life or availability of intact-polyphenols in plasma post dietary intervention also explains why its impact is not as pronounced in vivo in comparison to in vitro assays that demonstrate a favourable mode of action. Variation in gut microflora enzymes responsible for polyphenol metabolism and interaction with other dietary components are some of the other factors that either might pronounce or alleviate the action of RB derived bioactive compounds.

In addition to the aforementioned factors, polyphenols and other bioactive compounds discussed in this review have been known to possess pro-oxidant properties. For example, studies have claimed that “antioxidants” have the potential to stimulate oxidative DNA damage [84]. Often these pro-oxidant effects are associated with bioactive compounds interacting with transition metal ions [85]. It has been argued that bioactive compounds exert their cyto-protective/antioxidant properties in the GI tract due to the high concentrations that can be present. However, unabsorbed metal ions also present in the GI tract can stimulate pro-oxidant activity. Nevertheless, a mild-degree of pro-oxidant status might be instrumental in increasing antioxidant activity and xenobiotic-metabolising enzymes [85].

Another key factor that is quite often under-investigated is the hermetic actions of dietary bioactive components. Hormesis is used to describe a phenomenon wherein a bioactive component is known to induce contradictory effects at different doses. The most common phenomenon demonstrated has been with dietary phytochemicals that have a beneficial effect at lower doses and toxic at higher concentrations. Though this has not been particularly reflective with regard to RB bioactive compounds, it is an important mechanism of action that needs to be considered when evaluating it in vitro bioactivity.

## 5. Conclusions

Current literature on RB derived from whole grain rice, which at present is an under-utilised food ingredient, has shown RB to be a rich source of many bioactive compounds that have protective properties. The free radical scavenging ability and disease preventive properties of RB have been attributed to the presence of compounds such as acylated steryl glucosides, flavonoids, resveratrol, γ-oryzanol, ferulic acid, policosanol, tocotrienol, hydroxycinnamic acid derivatives and several bioactive peptides. Dietary supplementation of these bioactive compounds have shown improvements in vascular inflammation, endothelial function, hypertension, hyperlipidaemia and hyperglycaemia by reducing free-radical-mediated oxidative stress owing to the synergistic effect of several RB derived compounds. Though RB has bioactive properties that have the potential to target specific metabolic pathways in vitro, factors such as bioavailability, microflora profile, enzymatic involvement and phytochemical metabolism need to be considered when translating findings *in vivo*. 

## Figures and Tables

**Table 1:** In vitro and in vivo cardioprotective role of rice bran-derived bioactive compounds.

Sample	Variety (Source)	Target Group	Study Design and Intervention	Effect	Reference
**Impact of rice bran derived bioactive chemical compounds**					
Leaf, RB, brown and polished rice grains	Leaf samples (Habataki and Nipponbare cultivars) Brown rice sample (Nipponbare and Koshihikari cultivars) Bran and polished rice (sourced from a local market in Japan)	Metabolome analysis (LC-MS) In vitro assay	Potential anti-inflammatory and antioxidant activity of the samples were examined	Flavonoids such as tricin, tricin 7-O-rutinoside and tricin 7-O-β-d-glucopyranoside were mainly present in the leaf and bran Tricin 4′-O-(erythro-β-guaiacylglyceryl) ether and isoscoparin 2″-O-(6‴-(E)-feruloyl)-glucopyranoside showed the most potent activity for inhibiting NO production and DPPH radical-scavenging, respectively	[28]
Feruloylated oligosaccharides from RB	Unknown	In vitro cell culture, RAW264.7 cells	RAW264.7 cells were incubated with different concentrations of feruloylated oligosaccharides that were induced with or without lipopolysaccharide (LPS) LPS Cytokines (TNF-α, IL-1β, IL-6, NO, and IL-10) and PGE2 were examined	↑ IL-10 ↓ TNF-α, IL-1β, IL-6 and NO ↓ PGE2	[29]
RB policosanol extract	Unknown (sourced from Bernas milling factory in Kuala Selangor, Malaysia)	In vitro animal, Sprague–Dawley rat platelets	Rat platelets were pre-treated with policosanol extract (125–1000 μg/mL) Acid phosphatase assay and modified Lowry determination method was utilised	↓ Platelet adhesion to collagen ↓ Cellular protein secretion	[30]
RB enzymatic extract	Unknown	In vivo animal, ApoE^−/−^ mice (n = 15)	23-week trial ApoE^−/−^ mice were fed a low-fat diet (13% kcal) or high-fat diet (42% kcal) supplemented with or without 1 or 5% RB enzymatic extract (*w*/*w*)	↓ Serum lipid profile ↓ Atherosclerotic plaque development ↓ Liver steatosis	[34]
RB enzymatic extract	Unknown	In vivo animal, ApoE^−/−^ mice (*n* = 5–15)	23-week trial ApoE^−/−^ mice were fed a high-fat diet or an isocaloric high-fat diet supplemented with 5% (*w*/*w*) RB enzymatic extract	↓ Total cholesterol and triglycerides ↓ Macrophage infiltration ↓ Plaque development	[18]
RB enzymatic extract	Unknown (sourced from Dr. Juan Parrado from University of Seville)	In vivo animal, Zucker rats (*n* = 7)	20-week trial Lean and obese Zucker rats were fed standard diet supplemented with or without 1% and 5% RB enzymatic extract	↑ NO ↑ eNOS protein ↑ Calcium-activated potassium channels expression ↓ O_2_^−^ production ↓ Microvascular inflammation	[35]
RB enzymatic extract	Unknown	In vivo animal, Zucker rats (*n* = 7)	20-week trial Lean and obese Zucker rats were fed standard diet supplemented with or without 1% and 5% RB enzymatic extract	↑ eNOS protein ↓ Endothelial dysfunction and vascular hyperreactivity ↓ Aortic iNOS and TNF-α expression ↓ O_2_^−^ production ↓ NADPH oxidase regulation	[36]
Resveratrol formulation with 5% quercetin and 5% RB phytate (commercially known as Longevinex)	Unknown	In vivo animal, Sprague-Dawley rats (*n* = 4–6)	1–3 months trial Rats were gavaged with either Longevinex or vehicle (5% quercetin plus 5% RB phytate) After 1 or 3 months, the rats were sacrificed and isolated working hearts were subjected to 30 min ischemia followed by 2 h of reperfusion	Improved aortic flow and left ventricular function ↓ Myocardial infarct size ↑ Survival signals via phosphorylation of Akt ↑ Formation of LC3-II (from LC3-I) and Beclin-1 ↑ Sirt1 (nuclear) and Sirt3 (mitochondria) ↑ Anti-apoptotic protein Bcl-2 ↓ Pro-apoptotic protein Bax	[31]
RB enzymatic extract	Unknown	In vivo animal, male Wistar rats (*n* = 50)	At 12 weeks, the rats were gavaged with RB enzymatic extract (10 g kg^−1^) Blood, plasma urine and faeces were collected Antioxidant effect was examined	Total of 25 ferulic acid metabolites were found in the plasma and urine. In the faeces, colonic metabolism led to simpler phenolic compounds O_2_^−^ production was eliminated	[37]
Navy bean and RB	Unknown (RB was sourced from US Department of Agriculture-Agricultural Research Service Dale Bumpers National Rice Research Centre)	In vivo human, children with dyslipidaemia (*n* = 38)	4-week trial Control = no navy bean or RB Test = 17.5 g/day cooked navy bean powder, 15 g/day heat-stabilized RB or 9 g/day navy beans and 8 g/day RB Several biochemical parameters were examined	After RB consumption: ↑ Several metabolites ↑ Diet-derived amino acids, phytochemicals (salicylate), and cofactors/vitamins (pyridoxal) After consumption of RB combined with navy bean: ↑ Lipid metabolites ↓ Carnitine	[32]
Acylated steryl glucosides (PSG)	Unknown	In vivo human, post-menopausal Vietnamese women (*n* = 60)	6-month trial Test group consumed 6 capsules/day containing 50 mg PSG Placebo group consumed 6 capsules/day containing corn oil	↓ Serum LDL ↓ TNF-α levels	[27]
Whole-grain cold breakfast cereal, dark bread, oatmeal, brown rice, popcorn, bran and germ	Unknown	In vivo human (*n* = 71,750 women and 42,823 men)	Follow up study from 1984 and 1986 through to 2010 Using a Cox proportional hazards model, whole grain consumption in relation to ischemic stroke was examined	Increased intake of whole-grain cold breakfast cereals and bran can lower the risk of ischemic stroke	[38]
**Impact of rice bran derived bioactive peptides**					
RB bioactive peptides	Unknown	In vitro cell culture, Human umbilical vein endothelial cell (HUVECs)	HUVECs were treated with RB bioactive peptides under H_2_O_2_ stimulation	↓ H_2_O_2_ induced cell morphology changes ↓ H_2_O_2_ induced cell apoptosis ↓ Protein levels of cleaved caspase-3 and p-p65	[39]
RB protein hydrolysate	Jasmine rice (Hom Mali 105)	In vivo animal, Male Sprague-Dawley rats (*n* = 16)	16-week trial Rats were fed either a standard chow and tap water or a high-carbohydrate and high-fat diet and 15% fructose solution For the final 6-weeks, rats were orally gavaged with RB protein hydrolysate (250 or 500 mg/kg/day)	↑ Plasma nitrate/nitrite level ↑ Aortic eNOS expression ↓ Hypertension ↓ Hyperglycemia ↓ Insulin resistance ↓ Dyslipidemia ↓ Aortic pulse wave velocity ↓ Aortic wall hypertrophy ↓ ACE inhibitory activity ↓ TNF-α ↓ Plasma malondialdehyde ↓ O_2_^−^ production ↓ p47^phox^ NADPH oxidase expression	[40]
RB protein hydrolysate	Jasmine rice (Hom Mali 105)	In vivo animal, Male Sprague-Dawley rats (n = unknown)	6-week trial Rat model of two kidney-one clip (2K-1C) renovascular hypertension were intragastrically administered with 50 or 100 mg kg^−1^ of RB protein hydrolysate or distilled water	↑ Endothelium-dependent vasorelaxation ↑ Plasma nitrate/nitrite ↑ eNOS protein ↓ Blood pressure ↓ Peripheral vascular resistance ↓ Plasma ACE ↓ O_2_^−^ formation ↓ Plasma malondialdehyde ↓ p47^phox^ protein	[41]
**Impact of rice bran oil**					
Esterified RB oil	Unknown	Fatty acid characterisation In vitro cell culture, RAW264.7 cells	Gas chromatography (GC) analysis of fatty acid methyl esters DPPH assay Cellular cytotoxicity, RNA extraction and real-time-PCR was conducted	↑ γ-oryzanol and ferulic acid ↑ DPPH radical scavenging activity and anti-inflammatory potential ↓ iNOS ↓ COX-2 mRNA expression ↓ Fatty acids	[42]
RB oil	Unknown (sourced from a local supermarket, Mysuru, India)	In vivo animal, male Wistar rats (*n* = unknown)	60-day trial The control diet contained groundnut oil AIN-93 diets supplemented with or without an unsaponifiable fraction of RB oil and additional γ-oryzanol added	Regular RB oil diet containing unsaponifiable fraction: ↓ Pro-inflammatory mediators like ROS (O_2_^−^ and NO), eicosanoids (PGE_2_, TXB_2_, LTB_4,_ and LTC_4_), cytokines (TNF-α and IL-6) and hydrolytic enzyme (collagenase, elastase and hyaluronidase) Physically refined RB oil diet: ↑ Anti-inflammatory mediators (IL-4 and IL-10)	[44]
Coconut oil, canola oil, and physically refined RB oil	Unknown (RB oil was sourced from TSUNO, Osaka, Japan)	In vivo animal, Experiment 1 8-week-old male F1B golden hamsters (n = 30) Experiment 210-week-old male F1B golden hamsters (n = 36)	Experiment 1 8-week trial Examination of plasma lipid, lipoprotein, and cholesterol metabolism Experiment 2 10-week trial Examination of early atherosclerosis	RB oil diet resulted in: ↑ Neutral sterol excretion with no effect on bile acid excretion ↑ Intestinal HMG-CoA reductase activity ↓ Hepatic HMG-CoA reductase activity ↓ Aortic fatty streak ↓ Plasma total cholesterol ↓ Cholesterol absorption ↓ LDL	[45]
Coconut oil with blended RB oil or sesame oil	Unknown (RB oil was provided by A.P. Solvex Limited, Dhuri, India)	In vivo animal, male Wistar rats (*n* = 6)	60-day trial Examination of thrombotic parameters after consumption of coconut oil with blended and interesterified with sesame oil or RB oil	↑ Prostacyclin/thromboxane ratio ↓ ADP and collagen-induced platelet aggregation	[47]
Palm oil, RB oil and coconut oil.	Unknown (sourced from Alfa One^TM^ Rice Bran Oil; Hansell Food Group)	In vivo human, healthy participants (*n* = 26)	16-month trial (August 2014 and December 2015) Single-blind, randomised cross-over study of atherogenic risk in normolipidaemic subjects after ingestion of isoenergetic meals with either palm oil, RB or coconut oil	Palm oil diet: ↑ Susceptibility to develop exaggerated chylomicron remnantaemia which may contribute to atherogenic risk RB or coconut oil diet: No effect	[48]
RB oil	Unknown (prepared by the Arian Top Noosh Company, Tehran, Iran)	In vivo human, hyperlipidemic participants (*n* = 50)	4-week trial Samples of fasting blood collected before and after supplementation with RB oil (30 g/day)	↓ Weight, body mass index, waist, and hip circumferences ↓ Total cholesterol, LDL, and the atherogenic ratio of total cholesterol/HDL	[49]
RB and sunflower oil	Unknown	In vivo human, hyperlipidaemia participants (*n* = 14)	3-month trial Participants consumed either RB oil or refined sunflower oil Serum lipids, anthropometry, dietary and physical activity patterns were examined	↓ Plasma total cholesterol ↓ Triglyceride levels	[50]
Blend (70:30) of RB and safflower oil	Unknown (sourced from Saffola^®^ Total, Marico Ltd., India)	In vivo human, hyperlipidemic participants (*n* = 80)	3-month trial Test group consumed blended RB and safflower oil (1 L/person/month) Lipid profile and inflammatory markers were assessed	↓ LDL ↓ Oxidized LDL ↓ Total cholesterol ↓ High sensitivity c-reactive proteins	[51]
RB and sesame blend (80:20)	Unknown (sourced from Adani Wilmar Limited, Ahmedabad, Gujarat, India)	In vivo human, mild-to-moderate hypertensive (*n* = 300) and normotensive subjects (*n* = 100)	60-day trial The study was divided into: - Normotensives treated with RB/sesame oil blend (*n* = 100) - Hypertensives treated with RB/sesame oil blend (*n* = 100) - Hypertensives treated with nifedipine (20 mg/d; *n* = 100) - Hypertensives treated with the combination of RB/sesame oil blend and nifedipine (20 mg/d; *n* = 100) - Blood pressure, anthropometric and biochemical measurements were conducted	Normotensives treated with RB/sesame oil blend: = Lipid profile Hypertensives treated with RB/sesame oil blend: ↓ Total cholesterol ↓ Triglyceride ↓ LDL ↓ HDL Hypertensives treated with the combination of RB/sesame oil blend and nifedipine: ↓ Blood pressure ↓ Total cholesterol ↓ Triglyceride ↓ LDL ↑ HDL	[53]
RB and safflower blend (80:20)	Unknown	In vivo human, hyperlipidaemia patients (*n* = 73)	3-month trial Double-blind, controlled, randomised parallel-group study Participants consumed either study oil (safflower and RB oil blend) or control oil (usual cooking oil) The lipid profile was examined monthly	↓ LDL levels for 82% of hyperlipidaemic patients	[52]

Key: ↑ Increase, ↓ Decrease, = No change.

**Table 2:** In vitro and in vivo anti-diabetic role of rice bran-derived bioactive compounds.

Sample	Variety (Source)	Target Group	Study Design and Intervention	Effect	Reference
**Impact of rice bran phenolics and other chemical compounds**					
RB extract γ-tocotrienol	Unknown	In vitro INS-1 cells In vivo Wistar rat model (*n* = unknown)	In vitro experiment: Low glucose (3.0 mM), glibenclamide (1 µg/mL), and ethanol was used as control RB extract concentrations tested were (0.032, 0.1, 0.32 and 1.0 mg/mL) In vivo experiment: Acute clinical trial Saline was used as control Rice bran oral gavage (10 mg/kg) γ-tocotrienol oral gavage (10 µg/kg) From blood collected, insulin and glucose parameters were evaluated	In vitro analysis: ↑Insulin release In vivo analysis: RB extract increased plasma insulin levels but did not influence blood glucose levels γ-tocotrienol decreased blood glucose but did not affect insulin release	[66]
Phenolic acid fraction derived from RB Commercially purified ferulic acid	Unknown	In vitro, enzyme assay In vivo animal, male T2DM C57BL/KsJ-db/db mice (*n* = 8)	In vitro experiment: α-glucosidase inhibitory activity In vivo experiment: 17-day trial Diet consisted of oral administration of ethyl acetate fraction (0.2 g/kg) or ferulic acid (0.05 g/kg) Blood analysis, hepatic glycogen content and glucokinase activity was examined	In vitro experiment: Ethyl acetate fraction was a potent α-glucosidase inhibitor followed by *p*-coumaric acid and ferulic acid In vivo experiment: ↑ Plasma insulin, hepatic glycogen synthesis, and glucokinase activity ↓ Blood glucose ↓ Total cholesterol and LDL	[67]
Driselase and ethanol fractions of RB	Unknown	In vivo animal, male stroke-prone spontaneously hypertensive rats (*n* = unknown)	Driselase (solid fraction) and ethanol fractions (liquid fraction) were extracted from RB using 70% ethanol Rats were placed on an AIN-93M-based control diet or supplemented with 60 g/kg of driselase and ethanol fractions of RB Several biochemical analysis was performed	Plasma biochemical parameters: ↓ Blood pressure ↓ Blood urea nitrogen/creatinine ratio ↓ Albumin ↓ Total cholesterol ↓ Triglyceride ↓ Glucose levels ↑ HDL/LDL ratio ↓ ACE inhibitory activity ↓ NO Urinary biochemical parameters: ↑ NO ↓ Creatinine ↓ Urea ↓ 8-hydroxy-2′-deoxyguanosine levels	[68]
Driselase treated fraction of RB Commercially purified ferulic acid	Unknown	In vivo animal, stroke-prone spontaneously hypertensive rats of Izumo strain (*n* = 6 per test group)	8-week trial The rats were fed an AIN-93M (control diet), AIN-93M supplemented with either driselase (60 g/kg) or ferulic acid (0.01 g/kg) Blood for glucose measurement was collected from the tail vein 30, 60 and 120 min before and after being fed glucose (1·8 g/kg body weight) via a gastric tube Plasma, liver and urine parameters and gene expression measured	Driselase diet: ↓ Plasma total cholesterol ↑ Plasma HDL/LDL ratio ↓ Plasma/liver triacylglycerol ↓ Plasma ACE inhibitory activity ↓ Plasma/urinary NOx levels ↓ Urinary 8-hydroxy-20-deoxyguanosine levels ↓ Liver total lipid ↑ Liver total cholesterol ↓ Blood pressure ↓ Albumin ↓ Glucose ↓ Insulin ↓ Incremental area under the curve glucose ↓ Blood urea nitrogen/creatinine ratio ↓ mRNA expressions several metabolic parameters involved in glucose and lipid metabolisms	[69]
Driselase treated fraction of RB	Unknown	In vivo animal, stroke-prone spontaneously hypertensive rats of Izumo strain (*n* = 4 per test group)	3-week trial Nuclear magnetic resonance spectroscopy was used to identify the active ingredient in driselase fraction The rats consumed either water (control), acute administration of adenosine (10 mg/kg) or chronic administration of adenosine (10 mg/L) Several biochemical parameters and gene expression was examined	The active compound in driselase fraction was identified as adenosine Adenosine diet: ↑ HDL ↓ LDL ↓ Blood pressure ↓ Total cholesterol ↓ Triglyceride ↓ Free fatty acid ↓ Glucose ↓ Blood urea nitrogen/creatinine ratio ↓ Albumin ↓ Leptin ↓ Hepatic total lipid, total cholesterol, and triglyceride ↑ Adiponectin ↑ Plasma NO Improvements in mRNA expression levels of genes involved in lipid and glucose metabolism	[70]
Fermented RB	Unknown (sourced from Sunbran Company, Tendo, Japan)	In vivo animal, stroke-prone spontaneously hypertensive rats of Izumo strain (*n* = 4 or 6 per test group)	Dual fermentation of RB was conducted using fungi and lactic acid bacteria Single-dose supplementation - Rats consumed either fermented RB, non-fermented RB or water (control) (2 g/kg) Chronic supplementation - 4-week trial - Rats consumed either 5% fermented RB, non-fermented RB or water (control) Biochemical analyses, oral glucose tolerance test, insulin tolerance test, ACE inhibitory activity and gene expression examination was conducted	Single-dose supplement: ↓ Systolic blood pressure ↓ Plasma glucose ↓ Plasma insulin Chronic supplement: ↑ Plasma adiponectin levels ↑ Hepatic p-AMPK/AMPK ratio and phosphorylation of p-AMPKα ↓ Blood pressure ↓ Hypertension ↓ Bodyweight gain ↓ Epididymal fat mass ↓ Glucose ↓ Insulin ↓ Total cholesterol ↓ Triglyceride ↓ LDL ↓ Leptin/adiponectin ratio ↓ Hepatic total lipid, total cholesterol, and total triglyceride ↓ mRNA expression of genes involved in liver gluconeogenesis and lipogenesis	[71]
RB and milled rice flour	Unknown	In vivo human, T2DM patients (*n* = 28)	12-week trial Treatment group (*n* = 17) was given 20 g of autoclave stabilised RB Placebo group (*n* = 11) was given 20 g of milled rice flour, sprinkled on their food or drink	RB treatment group: ↑ Adiponectin concentrations ↓ Postprandial glucose ↓ The area under the glucose curve ↓ HbA1c values ↓ Serum total cholesterol ↓ LDL ↓ Plasma FFA	[72]
**Impact of rice bran bioactive peptides**					
RB peptides	Unknown (sourced from SATAKE Co. Ltd., Higshi-Hiroshima, Japan)	In vitro assay	RB peptides were prepared using two commercial proteases, Umamizyme G and Bioprase SP Recombinant DPP-IV was synthesised from human adult kidney The bioactive peptides from RB were identified using gel filtration, HPLC, protein sequencing and LC-MS	Leu-Pro and Ile-Pro were potent DPP-IV inhibitors	[73]
RB protein hydrolysates	Jasmine rice (Hom Mali 105)	In vitro cell culture, HepG2 cells	Insulin resistance was induced in HepG2 cells with IL-6 or high glucose Regulation of insulin signalling in HepG2 cells was examined using PCR and western blot analysis	↑ AMPK phosphorylation involved in cellular energy homeostasis ↑ Glucose utilization of HepG2 cells IL-6 induced state: ↓ Degradation of IRS-1 and Akt phosphorylation ↓ STAT3 activation and SOCS-3 expression ↓ Phosphorylation JAK, STAT3 and SOCS-3 High glucose induced state: ↓ AMPK and Akt ↓ Derangement in lipogenic gene expression (SREBP-1c and FASN)	[19]
RB protein hydrolysates	Unknown (sourced from The Organic Agriculture Community Enterprise, Lopburi province, Thailand)	In vivo animal, male Sprague-Dawley rats (*n* = 7 each test group)	18-week trial RB protein hydrolysates were prepared using protease G6 enzyme Rats were fed a high carbohydrate-high fat diet, then orally gavaged with 100 or 500 mg/kg of RB protein hydrolysates, pioglitazone (an insulin sensitising agent as positive control) 10 mg/kg, or tap water (negative control) Fasting blood glucose, glucose tolerance, insulin resistance, lipid profiles, adiponectin, leptin and gene expression was examined	↑ Adiponectin ↑ Expression of PPAR-γ mRNA in adipose tissues ↑ IL-10 ↓ Retroperitoneal fat ↓ Bodyweight ↓ Fasting blood glucose ↓ Glucose tolerance test ↓ Total cholesterol ↓ LDL ↓ Triglycerides ↓ Elevated levels of insulin and HOMA-IR ↓ Leptin levels ↓ Expression of lipogenic genes (SREBF1 and FASN) ↓ Expression of inflammatory genes including Il-6, TNF-α, NOS-2, and MCP-1	[75]
RB enzymatic extract	Unknown	In vivo animal, obese Zucker rats and their lean littermates (*n* = 7 each per group)	20-week trial RB was modified by enzymatic hydrolysis using endoprotease mixture Obese Zucker rats and their lean littermates were fed standard diet (controls), 1% or 5% RB enzymatic extract Blood biochemical assays, glucose/insulin levels, hepatic triglycerides and total cholesterol were examined	Obese Zucker rats under RB enzymatic extract: ↑ Adiponectin levels ↑ Glucose ↑ HDL ↓ Total cholesterol ↓ Total cholesterol/HDL ratio ↓ Insulin ↓ Triglycerides ↓ Systolic blood pressure ↓ HOMA-IR index ↓ Pro-inflammatory values of NO Lean Zucker rats under RB enzymatic extract: ↑ Adiponectin levels ↑ HDL ↑ Total cholesterol ↑ Total cholesterol/HDL ratio ↑ Systolic blood pressure ↑ HOMA-IR index ↓ Glucose ↓ Insulin ↓ Triglycerides ↓ Pro-inflammatory values of NO	[76]
RB enzymatic extract	Unknown (sourced from the Enzymatic Production Technology Group of the Department of Biochemistry and Molecular Biology, School of Pharmacy, University of Seville Spain)	In vivo animal, obese Zucker rats (*n* = 6–8 per test group)	20-week trial RB was modified by enzymatic hydrolysis using endoprotease mixture Obese Zucker rats were fed standard diet (controls), 1% or 5% RB enzymatic extract and compared against lean Zucker rats Measurement of adipocyte size, RNA and protein extraction of adipose tissue was conducted	Obese Zucker rats under RB enzymatic extract: ↓ Bodyweight ↓ Overall visceral abdominal and epididymal tissue Gene expression of visceral abdominal adipose tissue: - ↓ Pro-inflammatory values of NO - ↓ TNF-α - ↓ IL-6 - ↓ IL-1 β - ↓ iNOS	[77]
RB fortified soymilk and sugar-free soymilk	Unknown (sourced from Sunstar group, Osaka, Japan)	In vivo human, healthy, Chinese males (*n* = 17)	Randomised, crossover, single-blind clinical trial Participants consumed either white bread (control), RB soymilk co-ingested with white bread or sugar-free soymilk co-ingested with white bread Blood collected at different time points from which glycemic and insulinemic index was examined	↓ Glycaemic index ↓ Glucose/insulin ratio	[78]
**Impact of rice bran oil**					
RB and soybean oil	Unknown	In vivo animal, male Wistar rats (*n* = 32)	4-week trial RB oil was extracted by the supercritical CO_2_ fluid extraction method Diabetes was induced via streptozotocin/nicotinamide injections T2DM rats were fed soybean oil (control diet without γ-tocotrienol or γ-oryzanol) and RB oil (test diets) containing 0, 10, and 15 g RB oil which consisted of 0, 35.2, and 52.8 g γ-oryzanol and 0, 60 and 90 mg γ-tocotrienol/100 g of RB oil respectively Blood, plasma, livers and faecal samples were collected and examined	RB oil diet (test) compared to the soybean oil diet (control): ↑ Faecal neutral sterol and bile acid excretion ↑ mRNA expression of enzymes associated with cholesterol metabolism ↓ Insulin/glucose ratio ↑ Plasma lipids i.e., palmitic acid ↑ Hepatic lipid, i.e., oleic acid ↓ Plasma/hepatic lipids, i.e., stearic acid, arachidic acid, linoleic acid, linolenic acid, eicosapentaenoic acid ↓ Plasma/hepatic triglyceride and total cholesterol ↓ LDL ↓ Non-esterified fatty acid	[80]
RB and soybean oil	Unknown	In vivo animal, male Wistar rats (*n* = 16)	5-week trial RB oil was extracted by the supercritical CO_2_ fluid extraction method Diabetes was induced via streptozotocin/nicotinamide injections T2DM rats were fed 15% soybean oil (control diet without γ-tocotrienol or γ-oryzanol) and 15% RB oil (test diet which consisted of 5.25 g γ-oryzanol and 0.9 g γ-tocotrienol/150 g of RB oil Blood, plasma and liver samples were collected and examined	RB oil diet (test) compared to the soybean oil diet (control): ↑ Excretion of faecal neutral sterols and bile acid ↑ Total SFA and MUFA in plasma and liver ↓ Total cholesterol/HDL ratio ↓ Non-esterified fatty acid ↓ The area under the curve for insulin ↓ Hepatic cholesterol	[79]
Groundnut oil, RB oil, and sesame oil	Unknown	In vivo animal, male Wistar rats (*n* = unknown)	Rats were placed under an AIN-93 diet supplemented with 10 wt% of groundnut oil, RB oil and sesame oil in the form of native and minor constituent-removed oils	RB and sesame oil: ↓ Serum and hepatic lipids, 8-hydroxy-2-deoxyguanosine, hepatic cytokines and eicosanoids in leukocytes Native RB and sesame oil: ↑ SREBP-2 ↑ PPAR-γ ↓ NF-κB p65	[81]
RB and soybean oil	Unknown	In vivo human, T2DM patients (*n* = 35)	5-week trial Randomised, single-blind, placebo, comparison study Participants consumed either: - Placebo diet (*n* = 17) consisting of 250 mL soybean oil-modified milk containing 18 g soybean oil daily - RB oil diet (*n* = 18) consisting of 250 mL RB oil-modified milk containing 18 g RB oil daily At weeks 0 and 5, anthropometric measurements, haematology assessments and oral-glucose-tolerance examinations were conducted	RB oil group: ↑ Glucose parameters (fasting, 2-h postprandial blood glucose concentrations and the area under the curve for postprandial plasma glucose) ↓ Total cholesterol ↓ LDL	[82]
Sunflower, canola and RB oil	Unknown	In vivo human, postmenopausal women with T2DM (*n* = 72)	8-week trial Single-centre, single-blinded and randomised control trial Participants consumed: - Balance diet with 30 g/day sunflower oil (control) - Balance diet with 30 g/day canola oil (test group) - Balance diet with 30 g/day RB oil (test group) - Anthropometric measurements, dietary intake assessment and biochemical assays were conducted	Canola and RB oil diet compared with sunflower oil control diet: ↑ HDL ↓ Total cholesterol ↓ Triglyceride ↓ LDL	[83]

Key: ↑ Increase, ↓ Decrease, = No change.
